# Supplementary material for: Predictors of Hepatitis B screening and vaccination status of young psychoactive substance users in informal settlements in Kampala, Uganda
Source: PLoS One. 2022 May 19;17(5):e0267953. doi: 10.1371/journal.pone.0267953 (PMC9119510; doi:10.1371/journal.pone.0267953)
Supplement: Attachment — Submitted filename: Response to comments-HBV 01-03-22_TS.docx [file pone.0267953.s002.docx]

**Response to comments**

We would le to appreciate the editor and the reviewers for their insightful feedback on the paper. We have used the feedback to improve the manuscript. Below s a point to point response to the issues raised during the review.

**Reviewer #1**

1. **Comment:** The present manuscript is a well-written cross-sectional study on predictors of hepatitis B vaccination and screening among young adults with a history of psychoactive substance use. Authors have found some significant associations for socioeconomic indicators and lack of knowledge. In general, the manuscript can be considered for publication after minor revisions.

**Response:** Thank you for appreciating our efforts. We have revised the manuscript as recommended

2. **Comment:** Background: This section can be shortened to 3-4 structured paragraphs in order to improve the flow of the background. There is some unnecessary information that can be removed, especially in paragraph 3, and the 4th and 5th paragraphs can be summarized and combined into one paragraph. Also, the first sentence of background should be grammatically revised.

**Response:**

3. **Comment:** Methods: There is no need to name all divisions and hospitals in Kampala here. The last sentence of scope and design and the second sentence of study population belongs to the background.

**Response:** Thank you. This paragraph has been revised (Page 6).

4. **Comment**: What is Khat? Please define.

**Response:** Khat has been defined (page 6, lines 148-149)

5. **Comment:** Results: Some figures and tables are located incorrectly in the manuscript. Please check. For example, the section “predictors of hepatitis B screening” does not represent the data of figure 2, while the section “Hepatitis B vaccination status” is describing figure 2. Also, table 2, 3, and 4 has been wrongly named in the text.

**Response:** Thank you. These have been changed (Page 12, line 271)

6. **Comment:** This sentence should be revised: “only 2.7% (21/768) had received two vaccine doses while only 2.7% (21/768) had completed the hepatitis B vaccination schedule of 3 doses.”

**Response:** Thank you. The sentence has been revised. Page 12 lines 277-278

7. **Comment:** In addition to the above, there are some minor grammatical and linguistic errors that needs proofreading.

**Response:** Thank you. We have proofread the paper and addressed the grammatical and linguistic errors.

**Reviewer #2**

1. **Comment:** Unable to locate where the data is available without restriction.
   **Response:** The data have been uploaded as a supplementary file
2. **Comment:** Please explain what is meant by the term “settlement”. Is it permanent housing? Temporary? How long do people stay and why? Do they live in groups? Families?

**Response:** The term settlement has been defined n the methods section. Page 6 lines 142-144277-27

3. Give a rationale for excluding those who have lived there <6 months.

**Response:** The rationale has been added. Since we used respondent-driven sampling, it would have been difficult for the peers to ascertain whether the prospective respondents actually lived in the selected informal settlements and or if they were actually substance users if they had lived there for less than 6 months.

4. Comment: Revision should address vaccine related policies. If vaccines are required/recommended in childhood, recall bias is a major concern and should be described in the limitations. Be certain to reference other studies related to vaccine recall.

**Response:** It is less likely that recall bias would be an issue since all the respondents were born before hepatitis B was integrated into the national immunization programme (2002) which requires newborns to receive a short at 6, 10 and 14 weeks. Nonetheless we acknowledge that recall bias could still be a challenge and has been included n the study limitations. Page 20 Lines 411-412

5. **Comment:** No reference is provided in the introduction for the statement “they are more likely to be unaware of their Hepatitis B status, and unvaccinated”. If there is no evidence to support it, delete the statement

**Response:** Reference has been provided for that statement. Page 5 lines 11-119

6. Comment: Are use of marijuana and alcohol associated with risk of hepatitis B? Please spend some time explaining the theoretical framework to help readers understand why you included all substance users and not just injection drug users.

**Response:** This has been explained on Page 6 Lines 151-153

7. Explain how multiple risk factors tend to co-occur, etc.
   The theoretical framework should also address how covariates were chosen. It’s not appropriate to let the model decide which variables to include- please explain the rationale and revise your models appropriately. Consider causal pathways and do not adjust for factors that lie in the pathway.

**Response:** The selection of covariates has been addressed. Page Lines 191-202. The models have been revised accordingly. Page 17 and Page 14

8. **Comment:** How many were excluded due to intoxication? Clarify how this is accounted for in the response rate.

**Response:** Given the nature of recruitment we dd not account for non-response. Prior to recruitment we explained to both the primary and secondary seeds the criteria for inclusion in the study. This reduced the chances of recruiting individuals who were not eligible to zero. This was further confirmed with the screening tool. Page 7 Lines 179-181

9. What time of day was data collection performed?

**Response:** Data were collected between 8:00 am and 6:00 pm throughout the data collection period. This has been included in the manuscript.

10. **Comment:** Please spell out what you mean by RDS and fully explain how potential respondents were approached. Tone down the use of the word “always” unless you have evidence to back up such a strong claim.

**Response:** RDS has been spelt out. The way potential respondents were approached s reported in our earlier publications. A reference has been given^[1]^. Page 7 Lines 177-17

We have toned down the use of the word “always”

11. **Comment:** Remove any language related to unweighted analyses from your description of the methods. This makes it sound like you have weighted data and chose not to use the weights. This is misleading for readers. Given the study population, it would not be possible/practical to weight the data, and this is the only rationale- do not site a statistical rationale.

**Response:** All language related to unweighted analyses has been removed.

12. **Comment:** Are females more likely to be screened because of pregnancy-related practices? Are pregnant women routinely screened for hepatitis B?

**Response:** True. Although it is not yet part of the standard of care, women are screened for HBV during pregnancy. This has been incorporated in the discussion. Page 18 Page Lines 363-365

13. Remove the word “variable” from figure 2.
    **Response:** The word “variable” has been removed from figure 2.
14. What is meant by “sensitization”?

**Response:** This has been replaced with “creating awareness.” Page 20 Line 421

1. Ssekamatte T, Kibira SP, Tetui M, Isunju JB, Mugambe RK, Wafula ST, et al. Do sexual expectancies and inhibitions predict high-risk sexual behaviours? Evidence from a cross-sectional survey among young psychoactive substance users in informal settlements in Kampala, Uganda. BMC Public Health. 2021;21(1):1-13.
