# Supplementary material for: Predictors of Hepatitis B screening and vaccination status of young psychoactive substance users in informal settlements in Kampala, Uganda
Source: PLoS One. 2022 May 19;17(5):e0267953. doi: 10.1371/journal.pone.0267953 (PMC9119510; doi:10.1371/journal.pone.0267953)
Supplement: Attachment — Submitted filename: Response to comments.docx [file pone.0267953.s003.docx]

**Response to comments**

**Journal Requirements:**

Please review your reference list to ensure that it is complete and correct. If you have cited papers that have been retracted, please include the rationale for doing so in the manuscript text, or remove these references and replace them with relevant current references. Any changes to the reference list should be mentioned in the rebuttal letter that accompanies your revised manuscript. If you need to cite a retracted article, indicate the article’s retracted status in the References list and also include a citation and full reference for the retraction notice.

Response: The comments have been checked for completeness and correctness

**Reviewer #3:**

1. Comment: Authors have tried to answer all comments from the reviewers and the manuscript is much improved, particularly the methods and results sections. Here are some additional minor comments:

Response: Thank you for acknowledging our efforts in responding to the comments. The minor comments are also appreciated.

2. The abstract contains many repetitive words and needs to be rewritten to sound good. There is no need to repeat the title in the abstract.

Response: The abstract has been revised to reduce repetitions. Pages 2-3 lines 40-71

3. As I mentioned before, the introduction is long and contain some unnecessary or unrelated information. Authors may try to be concise and focused on the main problem that the current study is going to address. Also, this section can be shortened into 4-5 paragraphs.

Response: Thank you. The introduction has been shortened to 5 paragraphs. Page 4-5 lines 74-118

4. In general, discussion is well-written but can also be more summarized and some sentences can be combined. There are terms like young psychoactive substance users or informal settlements in Kampala that repeats many times throughout the manuscript. Avoid repetitions like this: …vaccination status of young psychoactive substance users in informal settlements in Kampala. Among young psychoactive substance users living in urban informal settlements, we found... (Discussion-1st paragraph)

Response: The discussion has been shortened. Repetitions have been removed from the discussion and other parts of the manuscript. Page 17 lines 334-403
